# Regulatory T-cell deficiency leads to features of autoimmune liver disease overlap syndrome in scurfy mice

**DOI:** 10.3389/fimmu.2023.1253649

**Published:** 2023-09-25

**Authors:** Kaan Yilmaz, Stefanie Haeberle, Yong Ook Kim, Marvin J. Fritzler, Shih-Yen Weng, Benjamin Goeppert, Verena K. Raker, Kerstin Steinbrink, Detlef Schuppan, Alexander Enk, Eva N. Hadaschik

**Affiliations:** ^1^ Department of Dermatology, University of Heidelberg, Heidelberg, Germany; ^2^ Department of Dermatology, University Medical Center Mannheim, Medical Faculty Mannheim, University of Heidelberg, Mannheim, Germany; ^3^ Institute of Translational Immunology, University Medical Center of Johannes Gutenberg University Mainz, Mainz, Germany; ^4^ Department of Medicine, Cumming School of Medicine, University of Calgary, Calgary, AB, Canada; ^5^ Smart Healthcare Interdisciplinary College, National Taipei University of Nursing and Health Sciences, Taipei, Taiwan; ^6^ Institute of Tissue Medicine and Pathology, University of Bern, Bern, Switzerland; ^7^ Institute of Pathology and Neuropathology, RKH Klinikum Ludwigsburg, Ludwigsburg, Germany; ^8^ Department of Dermatology, University Hospital Muenster, Muenster, Germany; ^9^ Division of Gastroenterology, Beth Israel Deaconess Medical Center, Harvard Medical School, Boston, MA, United States; ^10^ Department of Dermatology, University Hospital of Essen, Essen, Germany

**Keywords:** regulatory T cells, Treg, scurfy mice, autoimmune liver disease, overlap syndrome, primary biliary cholangitis, autoimmune hepatitis

## Abstract

**Introduction:**

Scurfy mice have a complete deficiency of functional regulatory T cells (Treg) due to a frameshift mutation in the *Foxp3* gene. The impaired immune homeostasis results in a lethal lymphoproliferative disorder affecting multiple organs, including the liver. The autoimmune pathology in scurfy mice is in part accompanied by autoantibodies such as antinuclear antibodies (ANA). ANA are serological hallmarks of several autoimmune disorders including autoimmune liver diseases (AILD). However, the underlying pathogenesis and the role of Treg in AILD remain to be elucidated. The present study therefore aimed to characterize the liver disease in scurfy mice.

**Methods:**

Sera from scurfy mice were screened for ANA by indirect immunofluorescence assay (IFA) and tested for a wide range of AILD-associated autoantibodies by enzyme-linked immunosorbent assay, line immunoassay, and addressable laser bead immunoassay. CD4^+^ T cells of scurfy mice were transferred into T cell-deficient B6/nude mice. Monoclonal autoantibodies from scurfy mice and recipient B6/nude mice were tested for ANA by IFA. Liver tissue of scurfy mice was analyzed by conventional histology. Collagen deposition in scurfy liver was quantified via hepatic hydroxyproline content. Real-time quantitative PCR was used to determine fibrosis-related hepatic gene expression. Hepatic immune cells were differentiated by flow cytometry.

**Results:**

All scurfy mice produced ANA. AILD-associated autoantibodies, predominantly antimitochondrial antibodies, were detected at significantly higher levels in scurfy sera. CD4^+^ T cells from scurfy mice were sufficient to induce anti-dsDNA autoantibodies and ANA with an AILD-related nuclear envelope staining pattern. Liver histology revealed portal inflammation with bile duct damage and proliferation, as in primary biliary cholangitis (PBC), and interface hepatitis with portal-parenchymal necroinflammation, as found in autoimmune hepatitis (AIH). In scurfy liver, TNFα and fibrosis-related transcripts including *Col1a1, Timp1, Acta2, Mmp2*, and *Mmp9* were upregulated. The level of proinflammatory monocytic macrophages (Ly-6C^hi^) was increased, while M2-type macrophages (CD206^+^) were downregulated compared to wildtype controls. Despite severe hepatic inflammation, fibrosis did not develop within 25 days, which is close to the lifespan of scurfy mice.

**Discussion:**

Our findings suggest that Treg-deficient scurfy mice spontaneously develop clinical, serological, and immunopathological characteristics of AILD with overlapping features of PBC and AIH.

## 1 Introduction

Regulatory T cells (Treg) represent a distinct subset of CD4^+^ lymphocytes which play a pivotal role in the maintenance of peripheral tolerance by actively preventing autoimmunity (1). The development and suppressive functions of Treg are essentially regulated by constitutive expression of the transcription factor Forkhead Box 3 (FoxP3) (2). As such, a disruption of the encoding gene gives rise to rampant expansion of autoreactive CD4^+^ T cells, which then infiltrate several organs, exacerbate and perpetuate tissue insult by recruiting other inflammatory cells. The consequence is a lethal systemic autoimmune disorder with multi-organ failure, as manifested by immune dysregulation, polyendocrinopathy, enteropathy, X-linked (IPEX) syndrome in humans and genetically equivalent *Foxp3* mutant scurfy mice (3, 4). Due to a complete deficiency in functional Treg, hemizygous male scurfy mice spontaneously develop a lymphoproliferative disease with systemic inflammation, particularly involving the skin, kidneys, lung, and the liver, and resulting in death within four weeks of age (5, 6). The loss of immune homeostasis does not only rely on T-cell dependent mechanisms but is also orchestrated by B cells via production of autoantibodies, including antinuclear antibodies (ANA) (7–11).

ANA represent serological biomarkers of a variety of systemic autoimmune disorders such as connective tissue disease (CTD) and autoimmune liver diseases (AILD) (12). Based on clinical, histopathological, and serological findings, three major immune-mediated liver diseases can be distinguished, i.e., autoimmune hepatitis (AIH), primary biliary cholangitis (PBC), and primary sclerosing cholangitis (PSC). Although most cases match criteria of one of these entities, features of multiple categories may rarely occur concomitantly within the spectrum of AILD, a clinical phenotype referred to as “overlap syndrome” (13). Despite well-defined diagnostic parameters, the current therapeutic armamentarium is predominantly limited to non-specific immunosuppression and/or anti-cholestatic agents. Hence, if insufficiently treated, chronic inflammation and protracted repair mechanisms in AILD can lead to liver fibrosis and ultimately to cirrhosis, the strongest predisposing factor for hepatocellular carcinoma (14, 15). Of note, liver dysfunction was also reported in patients with IPEX syndrome, which is mostly fatal within the first two years of life (16).

The precise pathogenesis underlying AILD and the specific role of Treg in these diseases are still elusive. Thus, the present study sought to characterize the hepatic disease spontaneously evolving in scurfy mice. In this context, we screened sera of scurfy mice for the existence of ANA by an indirect immunofluorescence assay (IFA), analyzed the staining patterns, and tested for autoantibodies against targets specific to or associated with AILD, i.e. antimitochondrial antibodies with three major epitopes (AMA-MIT3), valosin-containing protein/p97 (VCP), glycoprotein-210 (gp210), Kelch-like protein (KL), hexokinase (HK), lamin B1, liver cytosol type 1 (LC1), soluble liver antigen (SLA), liver kidney microsome (LKM), soluble protein 100 kDa (sp100), early endosomal antigen 1 (EEA1), Ge-1, glycine-trytophan protein of 182 kDa (GW-182), and argonaute protein (Ago2). We further examined histopathological alterations, fibrosis-related transcripts, and the cellular components of the inflammatory infiltrates in scurfy liver. Our findings indicate that Treg-deficient scurfy mice harbor clinical and serological features of AILD with overlapping characteristics of AIH and PBC.

## 2 Materials and methods

### 2.1 Mice

Female heterozygous *B6.Cg-Foxp3sf/J* (Scurfy) mice were purchased from Jackson Laboratories (Bar Harbor, ME, USA) and bred to *C57BL/6* wild-type (WT) male mice to generate hemizygous male *B6.Cg-Foxp3sf/Y* (scurfy) offspring. *B6.Cg-Foxn1nu/J* (B6/nude) mice were acquired from Jackson Laboratories. All mice were maintained under specific pathogen-free conditions at the central animal facility of the Interfaculty Biomedical Facility, University of Heidelberg, Germany. Animal work was performed in line with the animal protocols (35-9185.81/G-195/11, T13/16, and T58/16), approved by the animal care committee (Regierungspräsidium Karlsruhe).

### 2.2 Screening for antinuclear antibodies

Serum samples taken from scurfy and WT mice at day 21 of life were assessed for the presence of ANA by an IFA, as previously described (10). Briefly, sera were diluted in PBS with 0.2% Tween 20 (Roth, Karlsruhe, Germany) and were added to slides precoated with human epithelial cells (HEp-20-10) and primate liver tissue (Euroimmun, Lübeck, Germany). Goat anti-mouse IgG Alexa Fluor 488 (dilution 1:500, Invitrogen, Carlsbad, CA, USA) served as secondary antibody. For clarity, although some of the IFA patterns were clearly cytoplasmic, here we collectively refer to both cytoplasmic and nuclear patterns as ANA. IFA images were generated by a fluorescence microscope (Zeiss Axioscop 40, Carl Zeiss, Göttingen, Germany). Semiquantitative analysis was performed in accordance with the manufacturer’s recommendations. ANA titers ≥ 1:100 were considered positive. Morphological fluorescence patterns were classified and shown as designated from anti-cellular (AC) 0 (negative) to AC-29, according to the recently updated International Consensus on ANA Patterns (ICAP) (17, 18).

### 2.3 Detection of antigenic targets of ANA

A group of AILD-related autoantibodies (AMA-MIT3 (against PDC-E2, BCOADC-E2, and OGDC-E2), LKM, sp100, gp210, SLA, LC-1) were identified by Euroimmun Line Immunoassay (LIA; Euroimmun, Lübeck, Germany), as previously reported (19). Antibodies directed to HK and KL were measured by QUANTA Lite enzyme-linked immunosorbent assay (ELISA) (Inova Diagnostics Inc., San Diego, CA, USA) (20). Autoantibodies to GW182, Ago2, Ge-1, EEA1, VCP, and lamin B1 were detected using a laboratory developed multiplexed addressable laser bead immunoassay (ALBIA), as previously described (10, 19, 20). Briefly, 20 microliters (µls) of suspended beads bearing the covalently coupled antigen analyte, 25 μl of sample diluent (Inova Diagnostics Inc., San Diego, CA, USA) and 5 μl of diluted mouse serum were added into the wells of 96-well plate. The plate was incubated with agitation at 600 rpm for 30 min at room temperature (RT), followed by incubation in goat anti-mouse IgG phycoerythrin conjugated secondary antibody (0.5 μg/ml, Jackson ImmunoResearch Lab. Inc., West Grove, PA, USA) for 30 min and 600 rpm in the dark. Plates were analyzed by using a Luminex-100 plate reader (Luminex Corp., Austin, TX, USA). Cutoff levels were determined on positive and negative controls in each run and were set at three standard deviations (SD) above the mean for WT mice.

### 2.4 Hybridoma generation

Prior to the fusion, six scurfy mice were selected based on the presence of ANA by IFA. Total lymph node and splenic cells were pooled and fused with the murine myeloma cell line Sp2/0 (ATCC, Manassas, VA, USA), according to the standard protocol (21). Fused cells were selected by using hypoxanthine-aminopterin-thymidine (HAT) and hypoxanthine-thymidin (HT) medium (Sigma-Aldrich, St. Louis, MO, USA), and supernatants were screened for ANA on HEp-20-10 and primate liver tissue by IFA. ANA positive clones were chosen and grown in Dulbecco’s modified Eagle’s medium (DMEM; Lonza, Verviers, Belgium) and subcloned repeatedly to assure monoclonality.

### 2.5 Passive transfer of CD4^+^ T cells into *nu/nu* mice

CD4^+^ T cells were isolated from lymph nodes and spleens of scurfy mice and WT controls through magnetic activated cell sorting with CD4 MicroBeads (Miltenyi Biotec, Bergisch Gladbach, Germany). CD4^+^ T cells (2 x 10^6^; purity >95%) in 100 µL of PBS were transferred into 4- to 6-week-old B6/nude mice via tail vein injections, as previously reported (11). Four weeks after injection, monoclonal antibodies from hybridoma supernatants derived from splenocytes of recipient B6/nude mice were subjected to IFA to measure the production of ANA and anti-dsDNA autoantibodies using HEp-20-10/primate liver tissue and *Crithidia luciliae* substrate (1:10 dilution; Euroimmun, Lübeck, Germany), respectively. Fluorescence intensity was scored as follows: 0, no specific staining; 1, weakly positive staining; 2, intermediate positive staining; 3 strongly positive staining, as described previously (9).

### 2.6 Histologic analysis of hepatic inflammation

Liver tissues were obtained from scurfy mice and WT littermates at day 21 of life during routine necropsies and fixed in 4% neutral buffered formalin at 4°C overnight and then embedded in paraffin. 5 µm thick sections were stained with hematoxylin and eosin (H&E), according to standard protocols. For histological evaluation, periportal/periseptal interface hepatitis and portal inflammation were scored using the following criteria, originating and adapted from the modified Histological Activity Index (HAI) grading system by Ishak et al. (22): For periportal/periseptal interface hepatitis; grade 0 (absent), grade 1 (mild; focal, few portal areas), grade 2 (mild/moderate; focal, most portal areas), grade 3 (moderate; continuous around <50% of tracts or septa), grade 4 (severe; continuous around >50% of tracts or septa). For portal inflammation; grade 0 (none), grade 1 (mild; some or all portal areas), grade 2 (moderate; some or all portal areas), grade 3 (moderate/marked; all portal areas), grade 4 (marked; all portal areas). The final inflammation grade (0-4) was dictated by the higher score of both categories.

### 2.7 Collagen quantification

75 mg of liver tissue were taken from the left lobe between 15 and 25 days of life. Samples were hydrolyzed in screw-capped polypropylene tubes (Greiner Bio-One, Frickenhausen, Germany) in 6 N HCl (1.25 mL per liver) at 110°C overnight, followed by centrifugation to remove solids and the supernatant collected. Triplicates of 5 µl of the supernatant were placed in a 96 well-plate and mixed with 50 µl of 0.1 M citrate buffer, pH 6.0, and 100 µl of 150 mg chloramine T dissolved in citrate buffer (0.1 M, pH 6.0) for a 30-min incubation at RT. Next, 100 µl of Ehrlich´s (1.25 g of dimethyl-benzaldehyde dissolved in distilled water) was added to the reaction mixture and incubated at 65°C for 30 min. Absorbance was measured at 550 nm in an Infinite M200Pro spectrophotometer (Tecan, Crailsheim, Germany). A standard curve using L-hydroxyproline standard (Merck, Darmstadt, Germany) was prepared to determine hydroxyproline (HYP) concentration (23).

### 2.8 Real-time quantitative PCR

Total RNA was isolated using the RNeasy Mini kit (Qiagen, Hilden, Germany) and 1 µg of total RNA was reverse transcribed into cDNA with the qScript cDNA SuperMix (Quanta Biosciences, Gaithersburg, MD, USA) according to the manufacturer’s recommendations. Quantitative real-time PCR (qRT-PCR) was conducted using validated Taqman gene expression sets for mouse procollagen α1(I) (COL1A1) (Mm00801666_g1), α-smooth muscle actin (α-SMA, ACTA2; Mm00725412_s1), tissue inhibitor of metalloproteinases 1 (TIMP-1; Mm01341361_m1), transforming growth factor beta 1 (TGFβ1; Mm01178820_m1), tumor necrosis factor α (TNFα; Mm00443258_m1), interferon-γ (IFNγ; Mm01168134_m1), matrix metalloproteinase 2 (MMP-2; Mm00439498_m1), matrix metalloproteinase 9 (MMP-9; Mm00442991_m1), and matrix metalloproteinase 13 (MMP-13; Mm00439491_m1) (Life Technologies, Carlsbad, USA) on a Step One Plus Real-Time PCR System (Life Technologies, Carlsbad, CA, USA). Beta-2 microglobulin (*B2m*) served as an endogenous control for internal normalization (24). Data were analyzed using the ΔΔ-Ct method, as described (25). The fold change was calculated as 2-ΔΔCt.

### 2.9 Isolation of hepatic non-parenchymal cells

Liver tissues were obtained from scurfy mice and WT controls between 15 and 25 days of life. Livers were minced and cells were treated with collagenase buffer (0.4% collagenase type IV, Sigma-Aldrich, St. Louis, MO, USA), 154 mM NaCl, 5.6 mM KCl, 5.5 mM glucose, 20.1 mM HEPES, 25 mM NaHCO_3_, 2 mM CaCl_2_, 2 mM MgCl_2_, 1.6 nM DNase I (Applichem, Darmstadt, Germany) (pH 7.4) and dispersed with a gentle MACS dissociator (Miltenyi Biotec, Bergisch-Gladbach, Germany). Homogenates were incubated at 37°C for 30 min, passed through a 100 μm cell strainer (BD Bioscience, San Jose, CA, USA) and centrifuged at 21 ×g for 4 min in ice cold PEB buffer (PBS, 2 mM EDTA, 0.5% BSA). Supernatants were centrifuged at 300 ×g for 10 min and cell pellets were resuspended in PEB buffer. Red blood cells were lysed by adding 10 volumes of 150 mM NH_4_Cl, 10 mM KHCO_3_, 1 mM EDTA·2Na. Non-lysed immune cells were washed twice and suspended in PEB buffer, as previously described (26).

### 2.10 Flow cytometry

Non-parenchymal liver cells were blocked with the 2.4G2 anti-Fc receptor antibody (BD Bioscience, San Jose, CA, USA) and stained with antibodies recognizing CD11b, CD11c, CD45, F4/80, Gr-1, Ly-6C (BD Bioscience, San Jose, CA, USA; Biolegend, San Diego, CA, USA; eBioscience, San Diego, CA, USA). For intracellular staining, cells were fixed in Fix/Perm buffer (BD Bioscience, San Jose, CA, USA), washed in PBS containing 2% goat serum and incubated in Perm/Wash buffer (BD Bioscience, San Jose, CA, USA) with an anti-CD206 antibody serving as M2 macrophage marker (eBioscience, San Diego, CA, USA). Cell acquisition was conducted on a fluorescence-activated cell sorting (FACS) Canto II (BD Bioscience, San Jose, CA, USA) and analyzed with the FLOWJO software (TreeStar, Ashland, OR, USA), as previously reported (26). The gating strategy was as follows: Neutrophil subsets were enriched from viable CD45^+^ and Ly6G^+^ immune cells. Total hepatic macrophages were obtained by subsequent enrichment of viable subset of CD45^+^ and Ly6G^-^ immune cells and were separated into monocytic and resident macrophage subsets by gating separately for CD11b and F4/80. Monocytic macrophages from CD11b^hi^ F4/80^int^ subset were again subdivided regarding their expression of Ly-6C. Resident macrophages from CD11b^int^ F4/80^hi^ subset were further subdivided by the expression of CD11c and CD206 (**Supplementary Figure 1A**).

### 2.11 Statistical analysis

Results are expressed as mean ± SD. Differences were analyzed by Mann-Whitney U test, if not indicated otherwise. Significance was determined using Prism (GraphPad Software, La Jolla, USA) and p < 0.05 were considered significant. (∗) represents p < 0.05, (∗∗) represents p < 0.01, (∗∗∗) represents p < 0.001 and (∗∗∗∗) represents p < 0.0001. Rank-based non-parametric trend analysis of independent samples was performed with Jonckheere-Terpstra test using R Statistical Software (v4.3.1, URL: https://r-project.org) via clinfun package, and the corresponding figure was created using the ggbeeswarm and ggpubr packages.

## 3 Results

### 3.1 Scurfy mice produce ANA against AILD-specific and related antigenic targets

**Figure 1:**
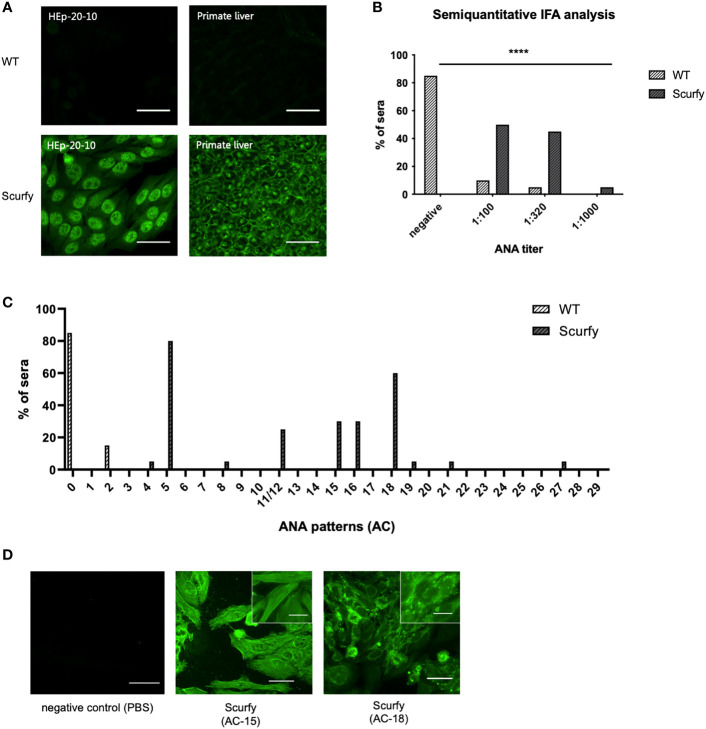
Scurfy mice produce ANA in the absence of Treg. **(A)** Representative images by IFA on HEp-20-10 cells and primate liver tissue with WT (upper panel) and scurfy (lower panel) serum. Scale bars, 50 µm. **(B)** Semiquantitative IFA analysis with ANA titers in sera of WT and scurfy mice ****p<0.0001 (Mann-Whitney U test). **(C)** Analysis of ANA patterns in WT and scurfy sera by using designated codes from AC-0 (negative) to AC-29, according to ICAP nomenclature (scurfy n=20, WT n=20). **(D)** Representative images by IFA on HEp-20-10 cells with hybridoma supernatants directly obtained scurfy mice. Cytoplasmic fibrillar linear pattern (AC-15, middle panel), cytoplasmic discrete dots/GW-body like pattern (AC-18, right panel), and negative control (left panel) are shown. Scale bars, 50 µm (larger images) and 20 µm (close-up views).

ANA are serological hallmarks of a wide range of autoimmune diseases including those that manifest AILD, most importantly AIH (27). Therefore, we initially screened sera of scurfy mice for the existence of ANA by IFA. All scurfy mice (n=20) produced ANA with end-point titers ranging from 1:100 to 1:1000, whereas ANA were found in only 15% of healthy littermates (3/20) (**Figures 1A, B**). In scurfy mice, the most frequently (in 80%) observed IFA staining pattern on HEp-20-10 cells was the nuclear coarse speckled pattern designated AC-5 according to ICAP nomenclature (17), followed by AC-18 (cytoplasmic discrete dots/GW body-like, in 60%) as well as AC-15 and AC-16 (cytoplasmic fibrillar linear and cytoplasmic fibrillar filamentous, in 30%, respectively) (**Figure 1C**). In the majority of scurfy sera, multiple IFA patterns occurred simultaneously (in 85%, data not shown), which suggests a polyclonal humoral response. In order to further distinguish specific patterns, hybridoma cell lines of activated plasma cells from scurfy mice (n=2) were generated and tested for ANA positivity until a singular IFA staining pattern was reached by repeated subcloning. Consistently, AC-15 and AC-18 patterns were detected (**Figure 1D**), which are found to be associated with AIH-1 and PBC, respectively (28, 29).

**Figure 2:**
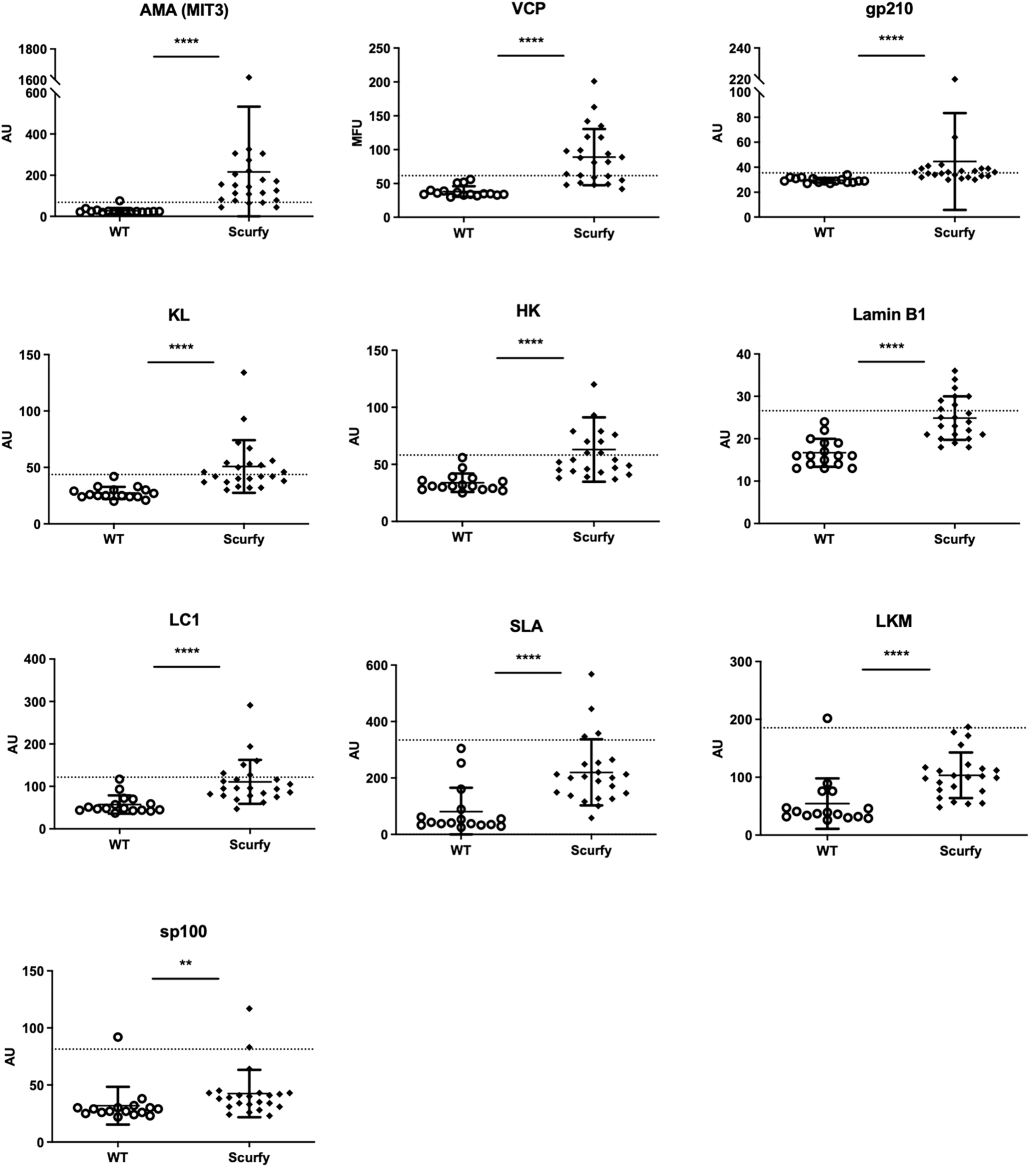
Scurfy mice exhibit AILD-associated autoantibodies. Detailed analysis of autoantibodies against antigenic targets associated with or related to AILD by using LIA, ELISA, and ALBIA. For ALBIA, titers are expressed in median fluorescent units (MFU). For LIA and ELISA, values are expressed in absorbance units (AU). Dashed lines represent cutoff values established at three SD over the mean of WT controls (scurfy n=23, WT n=16). **p<0.01, ****p<0.0001 (Mann-Whitney U test). Data are expressed as mean ± SD.

**Figure 3:**
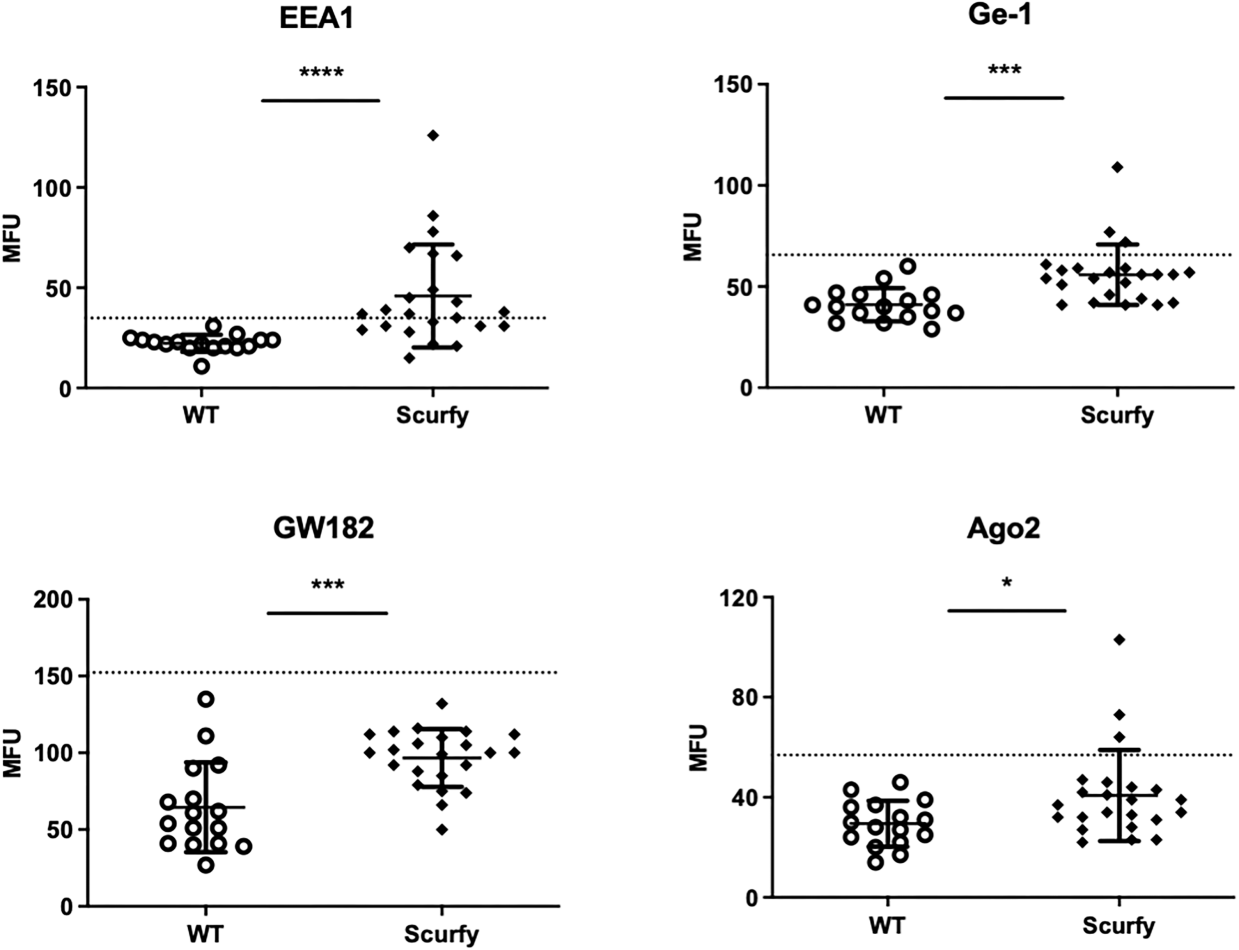
Production of autoantibodies against GW bodies in scurfy mice. Assessment of “cytoplasmic dot profile” by ALBIA in sera of WT and scurfy mice. Values are expressed in median fluorescent units (MFU). Dashed lines represent cutoff values established at three SD over the mean of WT controls (scurfy n=23, WT n=16). *p<0.05, ***p<0.001, ****p<0.0001 (Mann-Whitney U test). Data are expressed as mean ± SD.

As each AILD entity is characterized by a distinct serological profile, we subsequently determined the target antigens of AILD-specific autoantibodies. Interestingly, autoantibodies against all these targets were observed at significantly higher levels in scurfy sera compared with WT sera (**Figure 2**; **Supplementary Table 1**). AMA were the most prevalent autoantibody specificity in scurfy mice (in 82.6%), followed by anti-VCP (in 65.22%) and anti-gp210 autoantibodies (in 52.2%). Since AC-18 was the second most common pattern, we additionally performed a serological analysis for associated autoantibodies against EEA1, Ge-1, GW182, and Ago2 (“cytoplasmic dot profile”). Similarly, a significantly higher production was noted for all four autoantibodies in scurfy mice, especially for anti-EEA1 autoantibodies (**Figure 3**; **Supplementary Table 2**).

### 3.2 Autoreactive CD4^+^ T cells from scurfy mice induce ANA production with AILD-associated fluorescence pattern in B6/nude mice

**Figure 4:**
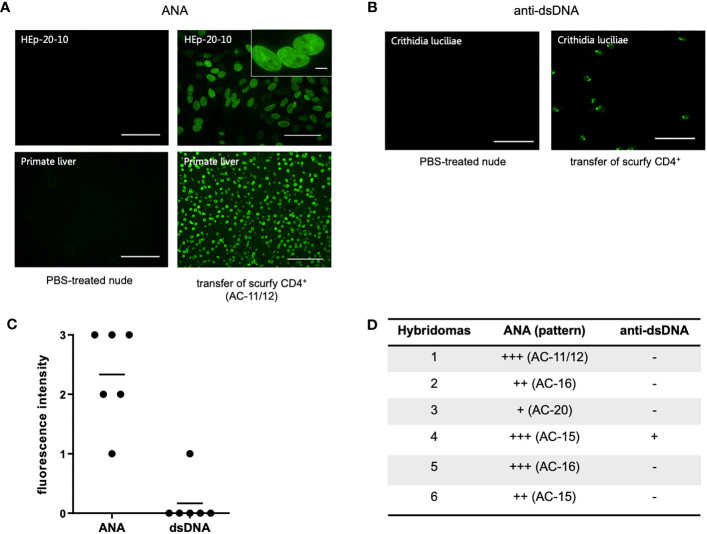
Transfer of scurfy CD4^+^ T cells induces production of ANA and anti-dsDNA autoantibodies in recipient B6/nude mice. **(A)** Representative images by IFA on HEp-20-10 cells and primate liver showing the nuclear envelope pattern (AC-11/12, right panel) in hydridomas from B6/nude mice after transfer of scurfy CD4^+^ T cells, while sera of PBC-treated nude mice were negative for ANA (left panel). Scale bars, 100 µm (left panel and right lower panel), 50 µm (right upper panel), 10 µm (close-up view). **(B)** Representative IFA staining on *Crithidia luciliae* substrate of monoclonal antibody from hybridomas derived from B6/nude mice transferred with scurfy CD4^+^ T cells, demonstrating anti-dsDNA positivity (right panel). No anti-dsDNA production was induced in nude mice by PBS injection (left panel). Scale bars, 50 µm. **(C)** Screening of monoclonal autoantibodies from hybridoma supernatants derived from splenocytes of recipient B6/nude mice for ANA and anti-dsDNA (n= 6). **(D)** Tabular summary of the IFA screening of hybridoma supernatants from recipient B6/mice after transfer of scurfy CD4^+^ T cells.

To assess if autoreactive CD4^+^ T cells are sufficient to induce production of AILD-related autoantibodies through T-cell dependent B-cell activation, CD4^+^ T cells of scurfy mice were adoptively transferred into T cell-deficient *nu/nu* mice. Remarkably, hybridoma supernatants obtained from splenocytes of all recipient *nu/nu* mice (n=6) revealed ANA positivity, whilst only one mouse showed anti-dsDNA autoantibodies (**Figures 4B–D**). Of note, one of the recipient mice yielded a nuclear envelope staining pattern (AC-11/12) (**Figure 4A**), which is strongly associated with AILD, particularly PBC (30).

**Figure 5:**
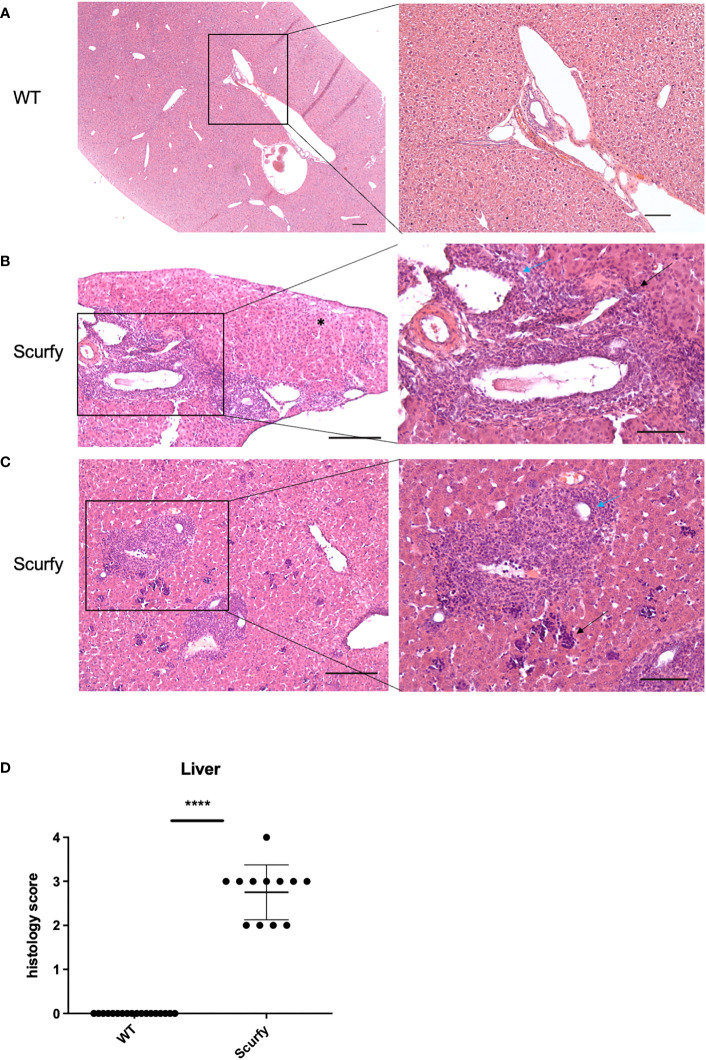
Scurfy mice spontaneously develop portal inflammation and interface hepatitis. **(A)** H&E staining of normal liver tissue of WT mice. **(B)** Severe lymphoplasmacytic infiltrates in enlarged portal tracts are shown. Necroinflammatory activity in hepatic parenchyma (asterix, left panel), interface hepatitis (black arrow, right panel), and lymphocytic infiltration of biliary epithelium of an irregular bile duct (blue arrow, right panel) were presented. **(C)** Marked inflammation of the portal tract including degeneration of the interlobular bile duct (blue arrow, right panel). Multifocal interspersed erythroblastic islands in hepatic parenchyma (black arrow, right panel). Scale bars, 200 µm (left panel) and 100 µm (right panel). **(D)** Grading of inflammation in scurfy and WT liver (scurfy n=12, WT n=18), ****p<0.0001 (Mann-Whitney U test). Data are expressed as mean ± SD.

### 3.3 Scurfy mice spontaneously develop portal inflammation with interface hepatitis and cholangitis

The histopathology of a liver biopsy is a major diagnostic criterion for most AILD as defined by disease-specific alterations of hepatocytes and/or biliary epithelium. Accordingly, we analyzed histomorphologic features of liver samples obtained from scurfy mice and WT controls. Notably, all scurfy mice revealed significant lymphoplasmacytic infiltration of enlarged portal tracts and degeneration and proliferation of interlobular bile ducts (**Figures 5B, C**), a feature typically seen in PBC. Moreover, AIH-like interface hepatitis and necroinflammatory activity in hepatic parenchyma were present (**Figures 5B, C**). Multifocal interspersed erythroblastic islands were also detected in hepatic parenchyma, indicating extramedullary hematopoiesis (**Figure 5C**), as previously reported (31). In line with previous findings (31), no granulomas were found. Overall, one scurfy mouse (8.33%) displayed a severe hepatic inflammation (grade 4), whereas grade 3 and grade 2 inflammation was observed in seven (58.33%) and four (33.33%) scurfy mice, respectively (**Figure 5D**). In contrast, no relevant inflammatory or degenerative anomalies were observed in the liver of WT mice (**Figure 5A**). A rank-based trend analysis revealed no significant correlation between serum levels of AILD-associated autoantibodies and histopathologic liver disease score in scurfy mice (**Supplementary Figure 2**), which might be attributed to the small sample size (n=12).

### 3.4 Profibrogenic transcripts are upregulated in the liver of scurfy mice

**Figure 6:**
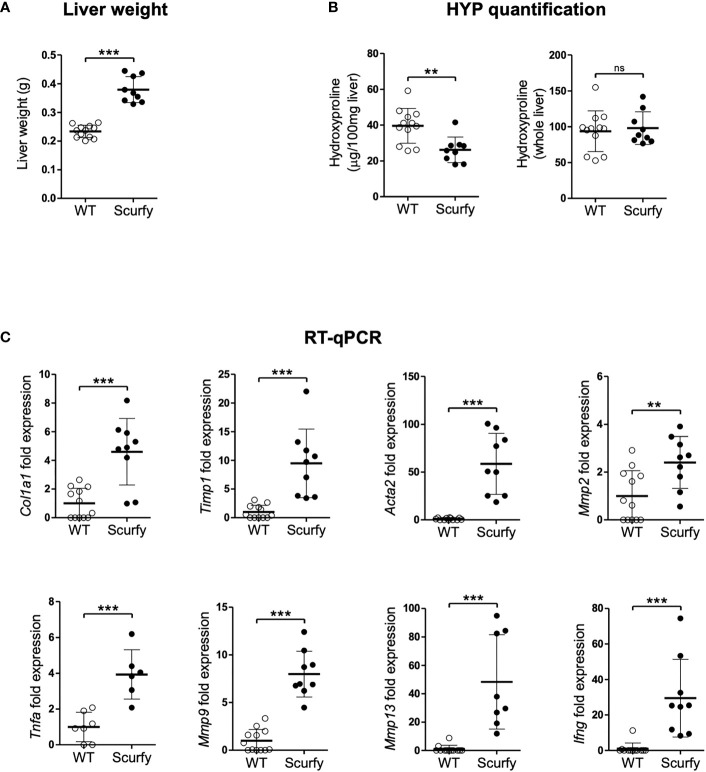
Upregulated profibrogenic transcripts in the absence of collagen accumulation in scurfy mice. **(A)** Assessment of liver weight in scurfy and WT mice. **(B)** HYP content per 100 mg liver (relative HYP, left panel) and per whole liver (total hepatic HYP, right panel) in scurfy and WT mice. **(C)** Levels of fibrosis-related transcripts in liver tissue of scurfy and WT mice (scurfy n=9, WT n=12). ns: not significant, **p<0.01, ***p<0.001 (unpaired Student's t-test). Data are expressed as mean ± SD. Results are representative of ≥2 independent experiments.

Liver fibrosis is characterized by the formation of a fibrous scar due to progressive accumulation of extracellular matrix (ECM) proteins, predominantly collagens (32). The gold standard to assess collagen deposition in fibrotic liver is through quantification of hydroxyproline (HYP), an amino acid unique to collagen (33, 34). In this regard, we measured HYP levels in scurfy und WT mice and found no significant changes in the total hepatic collagen content (**Figure 6B**), although the liver weights of scurfy mice were significantly increased (**Figure 6A**).

Subsequently, transcript levels of genes associated with hepatic fibrosis were assessed via qRT-PCR, which revealed a significant upregulation of fibrosis-related transcripts encoding proteins such as procollagen α1(I), TIMP-1, α-SMA (ACTA2), MMP-2, and MMP-9 in the livers of scurfy mice. Furthermore, a significant expression of TNFα was observed in the scurfy liver, contributing to the inflammatory environment in the hepatic tissue (**Figure 6C**).

### 3.5 Scurfy liver exhibits a proinflammatory phenotype with increased numbers of monocytic macrophages (Ly-6C^hi^)

**Figure 7:**
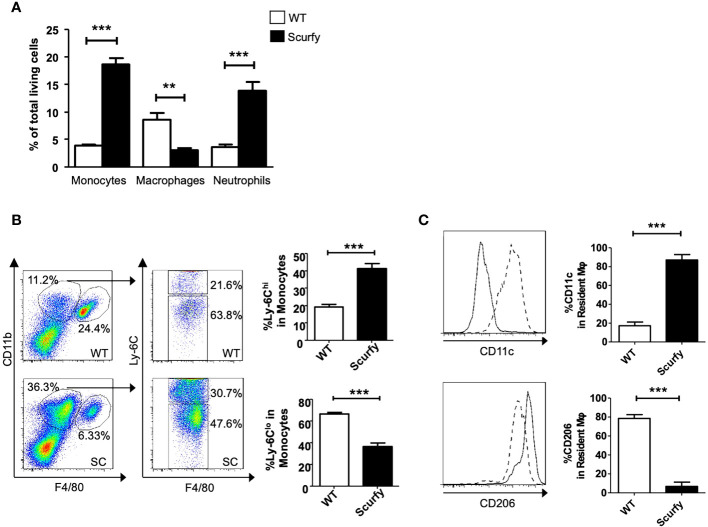
Increased pro-inflammatory and reduced restorative monocytes and macrophages in scurfy mice. **(A)** Bar graph indicating percentage of monocytes, macrophages and neutrophils in total living cells of WT and scurfy mice. **(B)** Representative FACS plots showing monocyte (CD11b^hi^ F4/80^lo^) and macrophage (CD11b^int^ F4/80^hi^) population (left panel). Monocytes were further analyzed by the differential expression of Ly-6C (right panel). Bar diagrams demonstrate the percentage of Ly-6C^hi^ and Ly-6C^lo^ population from monocytes. **(C)** Representative histograms showing the expression of CD11c and CD206 in macrophages. Solid line indicates WT mice; dot line indicates scurfy mice. Bar diagrams show the percentage of CD11c and CD206 in resident macrophages (*p<0.05, **p<0.01, ***p<0.001 unpaired Student’s t-test; scurfy n=6, WT n=6).

Monocyte-macrophages are not only key regulators in the maintenance, progression, and reversal of liver fibrosis, but also contribute to the pathogenesis of AILD (35). Therefore, we next focused on the cellular composition of the inflammatory infiltrates in liver of scurfy mice. Flow cytometry analysis revealed a proinflammatory phenotype in the absence of collagen accumulation. The number of monocytes and neutrophils was markedly elevated in scurfy liver tissue, whereas macrophages were detected at lower levels compared to WT controls (**Figure 7A**). Importantly, we identified increased inflammatory monocytic macrophages (Ly-6C^hi^) and reduced restorative monocyte populations (Ly-6C^lo^) in livers of scurfy mice (**Figure 7B**). Regarding liver-resident macrophages, increased proinflammatory M1 (CD11c^+^) and reduced M2 (CD206^+^) macrophages were detected in scurfy mice (**Figure 7C**; **Supplementary Figure 1**).

## 4 Discussion

AILD are immune-mediated chronic inflammatory disorders characterized by an abrogation of peripheral tolerance against hepatocytes and biliary epithelium (36). Screening for disease-related autoantibodies is essential to facilitate the diagnosis of AIH and PBC, whilst being of relatively minor importance in PSC. Although AIH is widely considered a T-cell mediated disease and the pathogenic role of autoantibodies remains ill-defined, several autoantibodies have been linked to different clinical phenotypes. As such, type 1 AIH (AIH-1, classic type) is associated with ANA and anti-SMA, whereas the rarer, yet more aggressive forms AIH-2 and AIH-3 are defined by the existence of anti-LKM/anti-LC1 and anti-SLA autoantibodies, respectively (12, 37–39). Considering the severe course of disease in scurfy mice, it is important to note that a significantly increased production of anti-LKM, anti-LC1, and anti-SLA autoantibodies was detected in sera of scurfy mice, along with ANA (**Figures 1**, **2**).

AMA, a serological hallmark of PBC, were by far the most prevalent AILD-associated autoantibodies in scurfy mice (in 82.6%, **Figure 2**), supporting previous findings (31). This is in accord with the predominant cholangitis in scurfy liver (**Figure 5**), while interface hepatitis and necroinflammatory activity in hepatic parenchyma, as seen in AIH, were observed to a lesser extent (**Figure 5**). Scurfy mice also revealed significantly elevated levels of anti-gp210 autoantibodies, which are reported to be associated with worse prognosis and higher risk at hepatic failure in PBC (40, 41). More recent evidence suggests that patients with anti-gp210 antibodies were more likely to develop interface hepatitis and lobular inflammation, akin to that in scurfy mice and PBC-AIH overlap syndrome (20, 31, 40). This concurs well with a study demonstrating that the prevalence of anti-gp210 autoantibodies was significantly higher in PBC-AIH overlap syndrome than in PBC and AIH (42). Furthermore, two recently identified biomarkers for PBC, anti-KL and anti-HK autoantibodies, were detected in almost half of scurfy sera (**Figure 2**). To date, little is known about clinicopathological correlations of these novel autoantibodies, although there is increasing evidence that anti-HK1 autoantibodies may be affiliated with poorer prognosis in PBC (43). Since cytoplasmic dot staining was frequently observed in scurfy sera by IFA, we opted for determination of autoantibodies against distinct cytoplasmic domains known as GW (G (glycine) W (tryptophan)-containing) bodies. These comprise unique cytoplasmic foci which exert critical functions in mRNA processing in the microRNA pathways and have been recently proposed as complementary biomarkers for PBC found in 5-10% of affected patients (19, 29, 44–46). In our study, scurfy mice yielded a significant production of autoantibodies against several GW bodies, i.e., GW182/TNRC6, Ago2, and Ge-1 (**Figure 3**). Interestingly, these structures do not possess a surrounding membrane, which may make them readily targetable by autoantibodies (19), as opposed to most of the other intracellular autoantigens in AILD which might only be released upon hepatocyte damage. By comparison, EEA-1 is part of the endosome/phagosome pathways with an IFA staining pattern that can resemble that of GW bodies, but comparatively little is known about its clinical associations (47).

Overlap syndromes are estimated to account for up to 20% of patients with PBC, the majority displaying features of both PBC and AIH (48, 49). Previous reports indicate that PBC-AIH overlap syndrome differs from isolated forms concerning disease course, prognosis, and therapeutic responses (50). Serologically, PBC-AIH overlap syndrome is generally defined by the presence of key autoantibodies of PBC and/or AIH, i.e. AMA and anti-SMA autoantibodies, and usually higher levels of transaminases and relative therapy resistance according to the Paris criteria (51). Recent studies, however, suggest that the serological profile of PBC-AIH overlap syndrome seems to be much more complex. Not only were other AILD-associated autoantibodies detected in this entity, including those against GW bodies, LKM, SLA, HK, gp210, KL, and sp100, but also distinct disease-specific serological patterns were reported (20). In this regard, anti-dsDNA autoantibodies have generated considerable interest since the concomitant existence of AMA and anti-dsDNA has been proposed to be highly specific for AIH-PBC overlap syndrome (52). This corroborates with our transfer experiments showing that autoreactive CD4^+^ T cells from scurfy mice were sufficient to activate B cells of recipient B6/nude mice to produce anti-dsDNA autoantibodies along with the AILD-associated AC-11/12 fluorescence pattern (**Figure 4**). Anti-dsDNA autoantibodies are typically regarded as serological biomarkers of systemic lupus erythematosus (SLE) (53). While it is assumed that autoantibodies in PBC-AIH overlap syndrome might target unique dsDNA epitopes, there is also evidence indicating an association between PBC and SLE as these entities overlap in some patients (20, 54). A higher incidence of CTD in AIH was also reported (55). We have previously shown that scurfy mice exhibit features of CTD, including SLE, scleroderma, and mixed connective tissue disease (9, 10, 56). Further studies are required to investigate the role of functional Treg deficiency as a common pathway in patients with concurring AILD and CTD. Given that anti-dsDNA autoantibodies were only detected in one out of six recipient B6/nude mice (16.67%) in the current study, the results should be interpreted with caution and need to be validated by future studies. Nevertheless, the proportion of B6/nude mice with anti-dsDNA production seems to reflect the proportion of autoreactive CD4^+^ T cells transferred from scurfy mice which are able to induce autoantibody production in the recipient B cell population, as anti-dsDNA autoantibodies were previously found to be prevalent in 15% of scurfy mice (10).

In this study, a wide range of AILD-related autoantibodies in sera of scurfy mice was detected (**Figures 2**, **3**). This finding can be explained by a polyclonal humoral response due to the uncontrolled expansion of CD4^+^ T cells in the absence of Treg, leading to production of more than a single autoantibody. On the other hand, the relationship between PBC and AIH in an overlapping setting is believed to be multilayered, ranging from sequential presentation of both entities, their simultaneous existence to being part of a disease continuum (57). As such, detection of multiple autoantibodies both in general and in the same patient has been reported in PBC-AIH overlap syndrome (20, 52). An important question to resolve for future research is why some autoantibodies (e.g., AMA, anti-VCP, anti-EEA1) were produced in the majority of scurfy mice, whereas others (e.g., anti-GW182, anti-LKM) were relatively rare.

As crucial effector cells of innate immunity, macrophages play an important role in the hepatic microenvironment via polarization to different phenotypes (classically activated M1-type and a spectrum of alternatively activated M2-type macrophages) under pathological conditions such as liver fibrosis, viral hepatitis, and hepatocellular carcinoma (35). The past five years have witnessed a renewed importance of macrophages in AILD as well. M1- and M2-type peribiliary macrophages were shown to be increased in human and murine PSC, while M1-type macrophages have been associated with enhanced Notch signaling and self-renewing phenotypes of hepatic progenitor cells (58, 59). In a concanavalin A (ConA)-induced AIH mouse model, splenectomy and IL-34 were found to drive M2 polarization which suppressed hepatic fibrosis and inflammation (60, 61). Furthermore, Li et al. demonstrated that cholangiocyte-derived exosomal long noncoding RNA H19 promoted M1 polarization and hepatic inflammation in PBC and PSC (62). These results indicate that M1 polarization seems to exacerbate AILD, whereas an M2-type polarization promotes inflammation resolution [reviewed in (35)]. This substantiates our findings in scurfy liver revealing a strong macrophage differentiation towards the M1 phenotype and a decreased M2-type polarization in a TNFα-dominated proinflammatory microenvironment (**Figures 6**, **7**) Surprisingly, this contrasts with our previous research which showed M2-polarized macrophages and a significant Th2 deviation in the skin of scurfy mice, as partly found in scleroderma (56). An explanation for these rather divergent responses may be that scurfy mice possibly recapitulate different stages of PBC-AIH overlap syndrome and CTD. This finding can also be attributed to potential differences in the cutaneous and hepatic inflammatory milieu. Further studies, including single-cell profiling, are needed to determine the heterogeneity and functional plasticity of macrophages in PBC-AIH overlap syndrome and organ-specific inflammation patterns in scurfy mice. Although a significant upregulation of profibrogenic or ECM remodeling transcripts was observed in scurfy liver, no collagen accumulation was detected (**Figure 6**). The finding that total hepatic collagen accumulation, as quantified by liver HYP, was not increased at sacrifice in scurfy mice, despite highly elevated fibrosis-related transcript levels, can be explained by their short lifespan, which necessitated their sacrifice at 15 to 25 days of age. Thus, only chronic inflammation of “wounds that do not heal” will lead from a Th1 T cell and M1-type fibrolytic immune cell response to a less inflammatory Th2 T cell and M2-type macrophage dominated response that drives tissue fibrosis (63–66). This would likely have been the case in scurfy mice if they would live longer than four weeks, as would be expected to occur in immune deficient patients (63–66). This is also in line with previous findings that fibrosis phenotypes, e.g., collagen accumulation, are significantly detected, chemically or macroscopically, after four weeks of age in most mouse models for liver fibrosis. In this context, at least four weeks are required for a statistically significant increase of collagen deposition in the carbon tetrachloride (CCL_4_)- and thioacetamide (TAA)-induced fibrosis model, which is the most representative mouse model for panlobular fibrosis (23, 67). An even slower fibrosis progression was noted for the biliary fibrotic *Mdr2* knockout mice which displays a significant hepatic collagen accumulation after ten weeks of age (23, 68). This observation was also reported in other age-dependent fibrosis development studies using genetically modified mouse strains in which the upward trend of hepatic HYP levels was clearer at four to eight weeks after birth (68). We therefore hypothesize that the early-onset lethal scurfy phenotype might represent a premature model for the full development of fibrosis and/or that their specific Foxp3 defect may favor both active fibrogenesis and fibrolysis, resulting in no significant net fibrosis. This can be supported by previous findings that MMP-9 and MMP-13, for instance, can be both fibrogenic via tissue remodeling and subsequent repair, but also be fibrolytic in other (resolution) settings (63, 69). Our findings further suggest a potential link between macrophages and CD4^+^ T cell-mediated tissue destruction in scurfy mice. In the aforementioned study by Haeberle et al., spontaneous Th2 cytokine secretion of skin infiltrating CD4^+^ T cells was associated with M2-polarized macrophages in the skin (56). Concordantly, scurfy mice were found to exhibit cartilage degradation and nonerosive arthritis in the paws, comprising CD3^+^ T lymphocytes, B cells, but also neutrophiles and macrophages (9). As previously demonstrated, in liver tissue of scurfy mice, CD4^+^ T cells were predominantly accumulated in periportal areas, while cytotoxic CD8^+^ T cells were concentrated around the bile ducts (31). The same study also identified increased expression of hepatic genes encoding cytokines such as IL-12, which is mainly produced by antigen-presenting cells including dendritic cells, monocytes, and macrophages (31, 70).

Previous reports indicate that, compared to isolated PBC, PBC-AIH overlap syndrome leads to significantly higher rates of unfavorable outcomes including esophageal varices, gastrointestinal bleeding, ascites, need for liver transplant, as well as abatement of 5-year survival (50, 71). Thus, there is an unmet need for therapeutic options which specifically target key molecules in the pathogenesis of AILD. Notably, a growing body of literature demonstrates safety and efficacy of adoptive Treg therapy in a variety of immune-mediated diseases (72, 73). However, available data on the precise role of Treg in AILD have been sparse and in part contradictory. In AIH patients, recent reports have suggested that Treg are fully functional and are not reduced in frequency (74, 75), despite initial studies indicating otherwise [reviewed in (73)]. Some researchers even found increased intrahepatic and peripheral Treg frequency in AIH, which was more prominent in pediatric patients than adults, implicating that intrahepatic Treg might be functionally defective or insufficient for disease control (75–77). Of note, a 4-year-old patient with IPEX syndrome was reported to develop AIH-2 with anti-LKM1 autoantibodies, which points to involvement of Treg in pathogenesis of AIH (78). In contrast, PBC has been clearly shown to be associated with functionally and numerically impaired Treg both in peripheral blood and liver of patients and mouse models (79–81). Further studies have highlighted the significance of Treg in PBC by demonstrating that deficiency in the alpha subunit of the IL-2 receptor (IL2RA; CD25) led to PBC-like liver disease in human und murine setting (82, 83). Importantly, healthy sisters and daughters of PBC patients were found to possess a significantly reduced Treg frequency, indicating a genetic susceptibility to Treg deficiency in PBC (84). In this context, our results might serve as an incentive for future research to determine the functionality and frequency of Treg in PBC-AIH overlap syndrome.

In summary, we confirm and extend previous findings on PBC-like liver disease in scurfy mice and provide further evidence of concomitant aspects typical of AIH. Our results support the hypothesis that Treg deficiency and the consequent breach of humoral tolerance in scurfy mice is key to the spontaneous development of clinical, serological, and immunopathological features of AILD with overlapping characteristics of PBC and AIH. The study sheds a new light on the role of Treg in the pathogenesis of immune-mediated liver disease.

## Data availability statement

The raw data supporting the conclusions of this article will be made available by the authors, without undue reservation.

## Ethics statement

The animal study was approved by Regierungspräsidium Karlsruhe, Germany. The study was conducted in accordance with the local legislation and institutional requirements.

## Author contributions

KY: Visualization, Writing – review & editing, Conceptualization, Data curation, Formal Analysis, Investigation, Methodology, Writing – original draft. SH: Conceptualization, Data curation, Formal Analysis, Methodology, Writing – review & editing, Investigation. YK: Visualization, Conceptualization, Data curation, Formal Analysis, Investigation, Methodology, Writing – review & editing. MF: Conceptualization, Data curation, Formal Analysis, Investigation, Methodology, Writing – review & editing, Supervision. S-YW: Visualization, Conceptualization, Data curation, Formal Analysis, Investigation, Methodology, Writing – review & editing. BG: Formal Analysis, Investigation, Methodology, Writing – review & editing. VR: Conceptualization, Data curation, Formal Analysis, Funding acquisition, Investigation, Methodology, Writing – review & editing. KS: Project administration, Conceptualization, Formal Analysis, Funding acquisition, Investigation, Methodology, Resources, Supervision, Writing – review & editing. DS: Formal Analysis, Project administration, Conceptualization, Funding acquisition, Investigation, Methodology, Resources, Supervision, Writing – review & editing. AE: Conceptualization, Funding acquisition, Investigation, Methodology, Resources, Supervision, Writing – review & editing. EH: Supervision, Writing – review & editing, Conceptualization, Formal Analysis, Funding acquisition, Investigation, Methodology, Project administration, Resources.

## Glossary

**Table:**

ACTA2 (α-SMA)	α-smooth muscle actin
ALBIA	addressable laser bead immunoassay
AC	anti-cellular
Ago2	argonaute protein
AMA-MIT3	antimitochondrial antibodies against PDC-E2, BCOADC-E2, and OGDC-E2
ANA	antinuclear antibodies
AIH	autoimmune hepatitis
AILD	autoimmune liver diseases
COL1A1	procollagen α1(I)
CTD	connective tissue disease
ECM	extracellular matrix
EEA1	early endosomal antigen 1
ELISA	enzyme-linked immunosorbent assay
FACS	fluorescence-activated cell sorting
FoxP3	Forkhead Box 3
gp210	glycoprotein-210
GW	G (glycine) W (tryptophan)-containing
GW-182	glycine-tryptophan protein of 182 kDa
HEp-20-10	human epithelial cells
HK	hexokinase
HYP	hydroxyproline
H&E	hematoxylin and eosin
ICAP	International Consensus on ANA Patterns
IFA	indirect immunofluorescence assay
IFNγ	interferon-γ
IPEX	immune dysregulation, polyendocrinopathy, enteropathy, X-linked
KL	Kelch-like
LC1	liver cytosol type 1
LIA	line immunoassay
LKM	liver kidney microsome
MMP	matrix metalloproteinases
PBC	primary biliary cholangitis
PSC	primary sclerosing cholangitis
qRT-PCR	quantitative real-time polymerase chain reaction
Treg	regulatory T cells
SLA	soluble liver antigen
SLE	systemic lupus erythematosus
sp100	soluble protein 100 kDa
TGFβ1	transforming growth factor beta 1
TIMP-1	tissue inhibitor of metalloproteinases 1
TNFα	tumor necrosis factor α
VCP	valosin-containing protein
WT	wildtype
