# Supplementary material for: Regulatory T-cell deficiency leads to features of autoimmune liver disease overlap syndrome in scurfy mice
Source: Front Immunol. 2023 Sep 25;14:1253649. doi: 10.3389/fimmu.2023.1253649 (PMC10561387; doi:10.3389/fimmu.2023.1253649)
Supplement: Supplementary file 1 [file DataSheet_1.docx]

Supplementary Material

Regulatory T-cell deficiency leads to features of autoimmune liver disease overlap syndrome in scurfy mice

Kaan Yilmaz, Stefanie Haeberle, Yong Ook Kim, Marvin J. Fritzler, Shih-Yen Weng, Benjamin Goeppert, Verena K. Raker, Kerstin Steinbrink, Detlef Schuppan, Alexander Enk, Eva N. Hadaschik^*^

*** Correspondence:** Eva N. Hadaschik; [Eva.Hadaschik@uk-essen.de](mailto:Eva.Hadaschik@uk-essen.de)

# Supplementary Figures


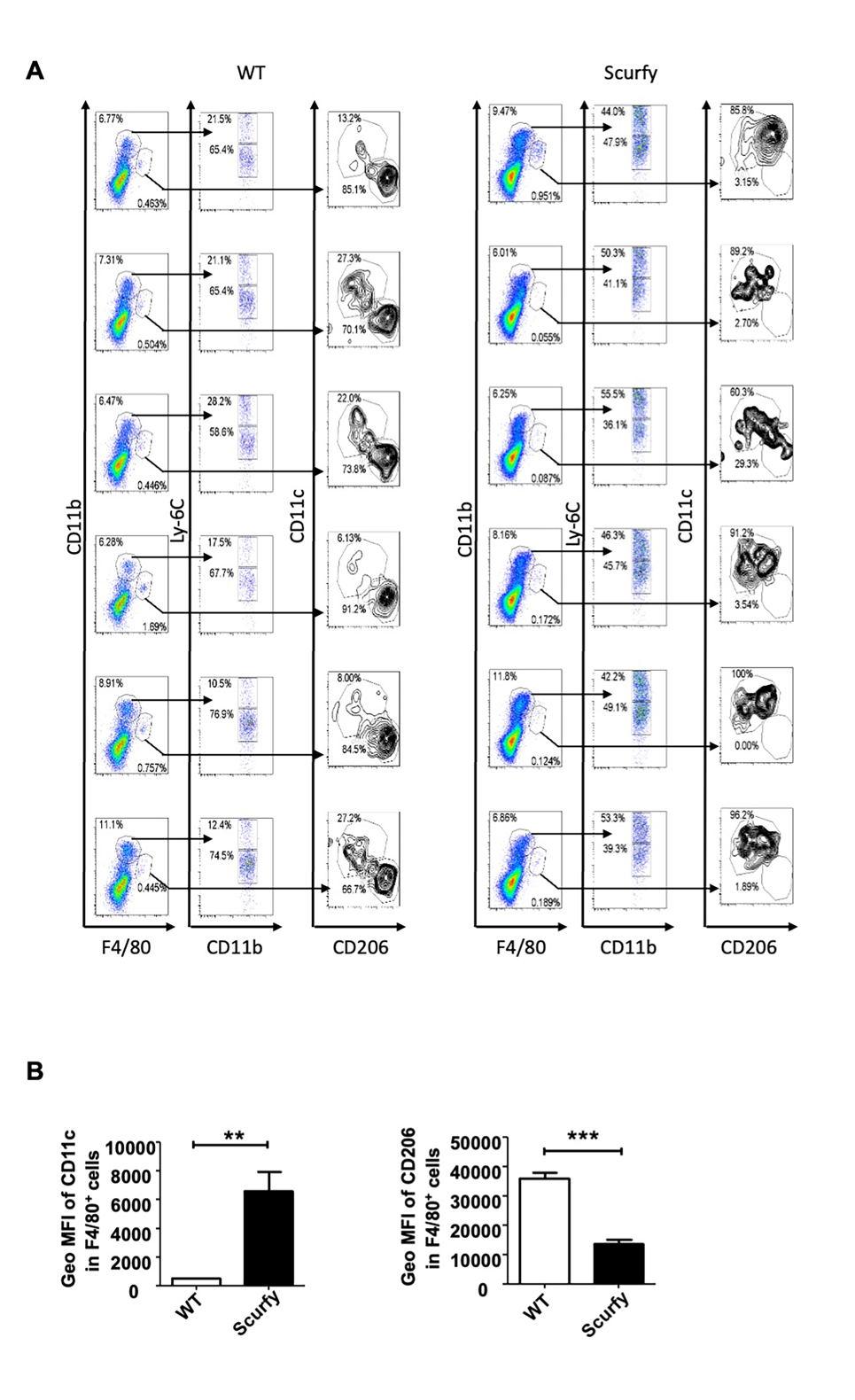


**Supplementary Figure 1.** **Analysis of hepatic immune cell profile of scurfy mice by flow cytometry.** (A) Gating strategy to identify various macrophage populations. Total hepatic macrophages were separated into monocytic and resident macrophage subsets by gating separately for CD11b and F4/80. Monocytic macrophages from CD11b^hi^ F4/80^int^ subset were further subdivided depending on their expression of Ly-6C. Resident macrophages from CD11b^int^ F4/80^hi^ subset were again subdivided by the expression of CD11c and CD206. (B) Representative bar diagrams showing the geometric mean fluorescence intensity (MFI) of CD11c and CD206 in macrophages (unpaired Student’s t-test; scurfy n=6, WT n=6).


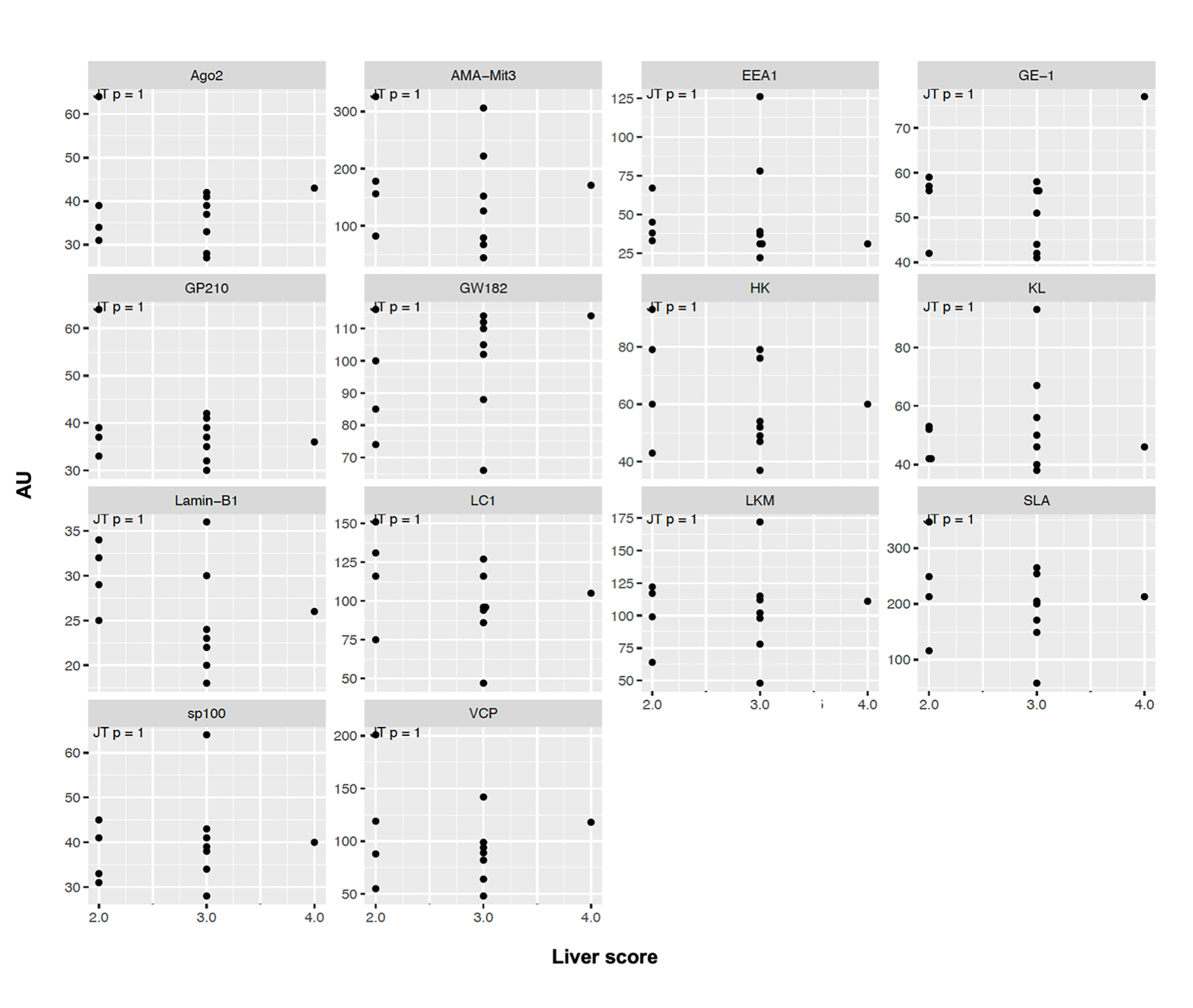


**Supplementary Figure 2.** **Evaluation of the correlation between AILD-associated autoantibody levels and histologic hepatic disease score in scurfy mice.** The x-axis represents the histologic liver score, while absorbance units (AU) are plotted on the y-axis in each graph (scurfy n=12; Jonckheere-Terpstra test).

# Supplementary Tables

**Supplementary** **Table 1. Serological profile of AILD-associated autoantibodies in scurfy and WT mice**

| **Antibody against/ associated with** | **Scurfy mice**  **(*n* = 23)** | | **WT mice**  **(*n* = 16)** | | **Cutoff** | **p value** |
| --- | --- | --- | --- | --- | --- | --- |
|  | **Units** | **+/-** | **Units** | **+/-** | **Units** |  |
| AMA (MIT3)^1^ | 216.3 ± 316.9 | 19/4 | 27.19 ± 13.77 | 1/15 | 68.5 | **** |
| VCP^2^ | 89.04 ± 41.46 | 15/8 | 38.19 ± 7.81 | 0/16 | 61.62 | **** |
| gp210^1^ | 44.57 ± 38.85 | 12/11 | 29.56 ± 1.97 | 0/16 | 35.47 | **** |
| KL^1^ | 50.78 ± 23.33 | 11/12 | 27.38 ± 5.43 | 0/16 | 43.67 | **** |
| HK^1^ | 63 ± 28.22 | 10/13 | 33.94 ± 8.05 | 0/16 | 58.09 | **** |
| Lamin B1^1^ | 24.87 ± 5.14 | 8/15 | 16.69 ± 3.3 | 0/16 | 26.59 | **** |
| LC1^1^ | 110.7 ± 51.77 | 6/17 | 57.31 ± 21.44 | 0/16 | 121.63 | **** |
| SLA^1^ | 219.9 ± 117.1 | 4/19 | 80.75 ± 84.53 | 0/16 | 334.34 | **** |
| LKM^1^ | 103.4 ± 39.53 | 1/22 | 54.5 ± 43.64 | 1/15 | 185.42 | **** |
| sp100^1^ | 42.52 ± 20.74 | 2/21 | 31.88 ± 16.5 | 1/15 | 81.38 | ** |

Values are expressed in ^1^absorbance units (AU) or ^2^median fluorescence units (MFU). Mann-Whitney U test; *p < 0.05, **p < 0.01, ***p < 0.001, ****p < 0.0001. Data are expressed as mean ± standard deviation (SD).

**Supplementary Table 2. Cytoplasmic dot profile in sera of scurfy and WT mice**

| **Antibody against/ associated with** | **Scurfy mice**  **(*n* = 23)** | | **WT mice**  **(*n* = 16)** | | **Cutoff** | **p value** |
| --- | --- | --- | --- | --- | --- | --- |
|  | **MFU** | **+/-** | **MFU** | **+/-** | **MFU** |  |
| EEA1 | 45.96 ± 25.65 | 14/9 | 22.38 ± 4.18 | 0/16 | 34.92 | **** |
| Ge-1 | 55.87 ± 14.95 | 3/20 | 41.06 ± 8.19 | 0/16 | 65.63 | *** |
| GW182 | 96.65 ± 18.78 | 0/23 | 64.56 ± 29.25 | 0/16 | 152.31 | *** |
| Ago2 | 40.74 ± 18.21 | 3/20 | 29.44 ± 9.14 | 0/16 | 56.86 | * |

Values are expressed in median fluorescence units (MFU). Mann-Whitney U test; *p < 0.05, **p < 0.01, ***p < 0.001, ****p < 0.0001. Data are expressed as mean ± standard deviation (SD).
